# Augmented Over-The-Top Technique for Revision Anterior Cruciate Ligament Reconstruction

**DOI:** 10.1016/j.eats.2025.103856

**Published:** 2025-09-04

**Authors:** Simone Perelli, Matteo Messori, Jesus Dueñas, Nicola Pizza, Rodolfo Morales Avalos, Maximiliano Ibañez, Joan Carles Monllau

**Affiliations:** a Institut Català de Traumatologia i Medicina de l'Esport (ICATME)-Hospital Universitari Dexeus, Universitat Autònoma de Barcelona, Barcelona, Spain; b Department of Surgery and Morphologic Science, Orthopaedic Surgery Service, Universitat Pompeu Fabra, Hospital del Mar, Barcelona, Spain; c ASST Grande Ospedale Metropolitano Niguarda, Milano, Italy; d Hospital Universitario Torrecardenas, Almeria, Spain; e Department of Traumatology, Medical Services of the Autonomous University of Nuevo Leon, Monterrey, Mexico

## Abstract

Revision anterior cruciate ligament reconstruction is technically demanding, particularly in cases with femoral tunnel malposition or tibial tunnel enlargement. The over-the-top (OTT) technique offers a single-stage solution by avoiding femoral tunnel drilling. However, its main limitation is the reduced graft diameter, typically between 5.5 and 7 mm, which may be insufficient in revision settings. This Technical Note describes an augmentation of the classic OTT technique using a peroneus longus tendon allograft wrapped around preserved-insertion hamstring tendons, achieving graft diameters exceeding 8 mm. Standard arthroscopic portals are used to prepare a standard tibial tunnel, whereas a lateral femoral approach is performed to prepare the OTT point. Femoral fixation is secured with staples, and a lateral extra-articular tenodesis is performed to enhance rotational stability. This combined intra- and extra-articular reconstruction allows single-stage revision even in the presence of tunnel enlargement, improving biomechanical strength and potentially accelerating biological integration through preservation of the tibial insertion. The described technique offers a reliable, reproducible, and cost-effective option for complex anterior cruciate ligament revisions.

The failure rate of anterior cruciate ligament (ACL) reconstruction attributed to biomechanical misalignment, improper graft integration, or inadequate rehabilitation^1^ ranges from 5% up to 25% within the first 10 postoperative years.^2^ When a revision of the ACL is needed in cases of a femoral tunnel malposition or widening, the over-the-top (OTT) technique can be useful because there is no need for a femoral tunnel.^3^ Lateral extraarticular tenodesis (LET) has been shown to be useful in ACL-deficient knee.^4^^,^^5^ This even more so in the setting of an ACL revision as persistent rotational instability often requires additional procedures like LET to increase knee stability.^6^ For this reason, the OTT technique is even more useful in the setting of a revision ACL reconstruction because both intra- and extra-articular procedures are made at the same time. Unfortunately, the intrinsic problem of OTT is the small diameter of the graft that normally varies between 5.5 and 7 mm in diameter. Although not problematic in primary ACL reconstruction, this may be a limitation in revision cases with tibial tunnel diameters exceeding 8 mm from the index procedure. The present work introduces a modification of the original OTT technique, as described by Marcacci et al.^6^ with the addition of a peroneus longus tendon allograft to increase the diameter of the graft. This modification permits to use the OTT technique in the setting of a revision surgery.

## Surgical Technique

### Patient Positioning

**Fig 1:**
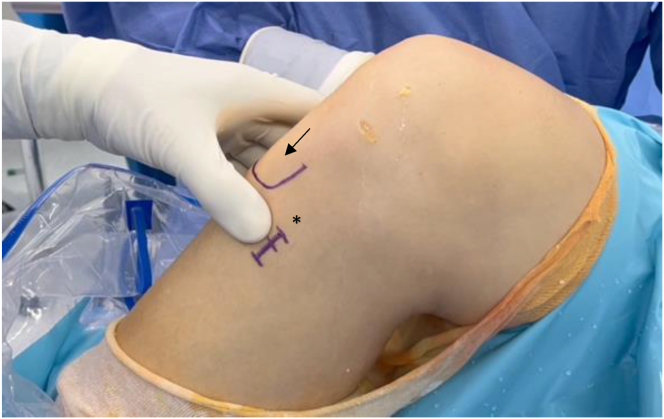
Patient is in the supine position, right knee flexed at 90°. A 2- to 3-cm longitudinal line is marked one finger medial to the tibial tuberosity (black arrow) and at the level of the fibular neck for hamstring tendon. Black asterisk denotes the incision line.

The patient is positioned supine. A tourniquet is placed, and the limb is supported by a lateral leg holder while keeping the knee at 90° of flexion (Fig 1).

### Hamstring Graft Harvesting

**Fig 2:**
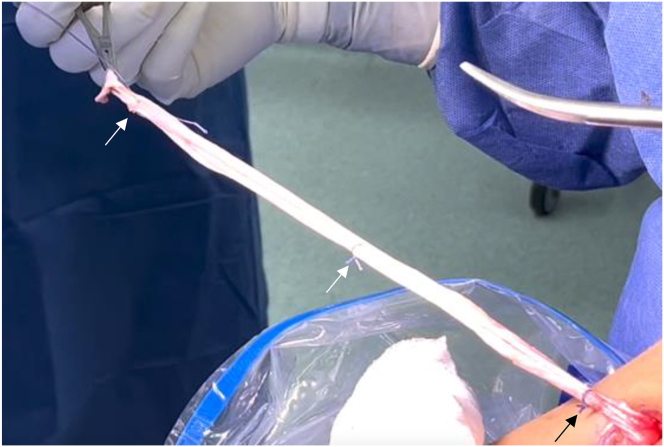
Once identified, hamstring tendons are stripped preserving their tibial insertion and unified with absorbable sutures (3 arrows).

A longitudinal incision of approximately 3 cm is made 2 cm medially to the anterior tibial tuberosity. After dissecting the subcutaneous tissue, the hamstring tendons are palpated, and a transverse incision is made through the sartorius muscle fascia just over the most proximal tendon. Using a 90° Semb dissecting clamp, both hamstring tendons are identified and extracted using an open tendon stripper preserving their tibial insertion. Once the soft tissue and muscle attachments are removed, both tendons are stitched together (Fig 2).

### Preparation of Peroneus Longus Allograft

**Fig 3:**
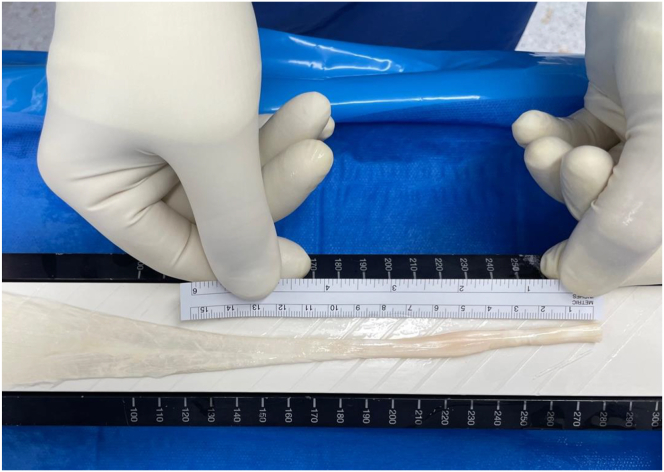
The peroneus longus allograft is measured and thawed for at least 10 minutes in a saline solution containing 1 g of vancomycin/100 mL before being opened in a sheet-like configuration. The length of the graft must match the length of the hamstring graft.

**Fig 4:**
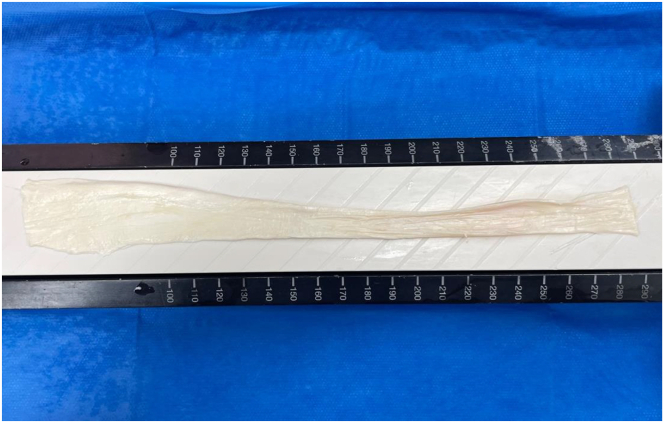
The peroneus longus allograft opened in a sheet-like configuration after longitudinal incisions in the distal half of the tendon.

A peroneus longus allograft is used (Fig 3). The graft is thawed for at least 10 minutes in a saline solution containing 1 g of vancomycin/100 mL to reduce the possibility of infection maintaining the mechanical quality of the tendon.^7^ A longitudinal incision is made along the distal half of the tendon, which has a more tubular shape. This portion of the tendon is opened in a sheet-like configuration (Fig 4).

### Augmentation of Hamstring Graft With Peroneus Longus Allograft

**Fig 5:**
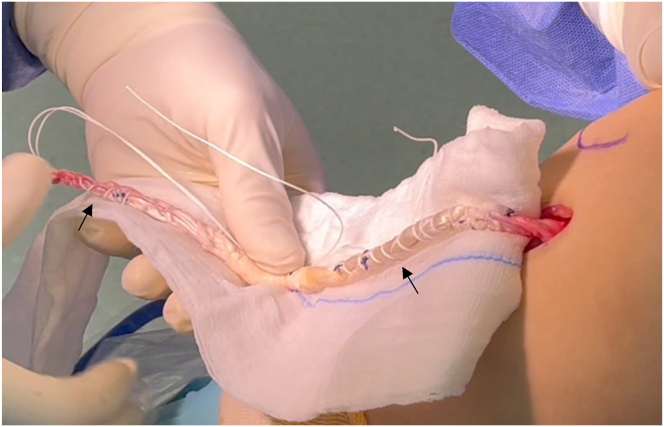
Final graft after unification and tubularization of the peroneus longus allograft around the hamstring autograft. Initial fixation is achieved using four simple absorbable sutures, followed by continuous high-strength sutures at both proximal and distal ends (black arrows).

The peroneus longus tendon is wrapped around the previously prepared hamstring tendons and firmly fixed around them using 4 simple absorbable sutures to start off with and is finished with continuous high-strength sutures (Fig 5). The graft is then protected with a gauze soaked in a saline solution containing 1 g of vancomycin/100 mL.

### Arthroscopic Stage

**Fig 6:**
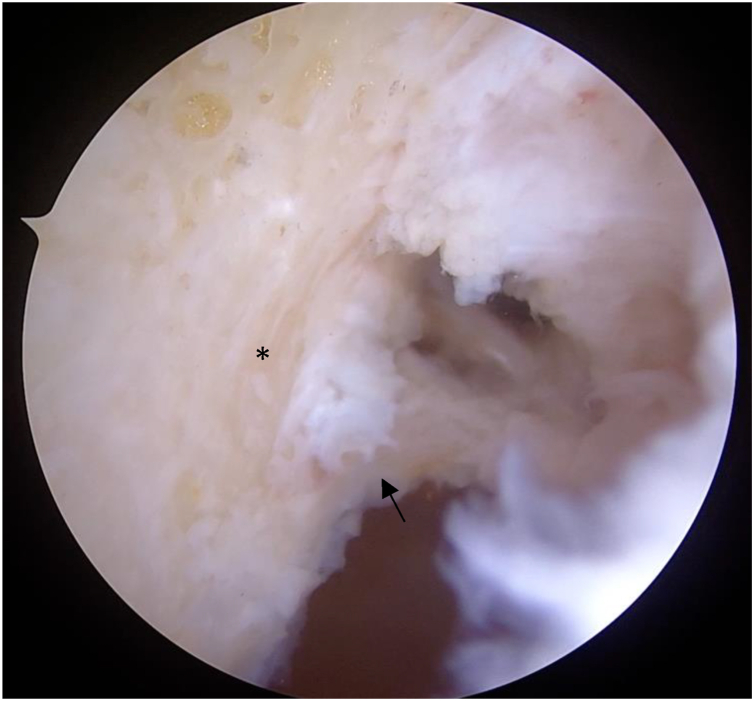
Arthroscopic view from the anteromedial portal after debridement of the previous graft with the knee flexed at 90°, showing the posterior aspect of the lateral femoral condyle (right knee; black asterisk). The black arrow indicates the previous anterior cruciate ligament footprint.

Standard arthroscopic exploration is performed through the anterolateral, anteromedial, and accessory anteromedial portals. The previous graft is debrided to allow optimal visualization of the posterior aspect of the lateral femoral condyle, extending to the OTT position (Fig 6). This area must be cleared of all soft tissues and, if needed, refreshed with a shaver or rasp to promote graft integration. Using the area just medial to the anterior horn of the lateral meniscus as a landmark, a 60° ACL aiming guide is introduced. The tibial tunnel is then drilled based on the previously measured graft diameter, and a dilator is used to ensure a stable tunnel wall.

### Lateral Femur Approach and OTT Preparation

**Fig 7:**
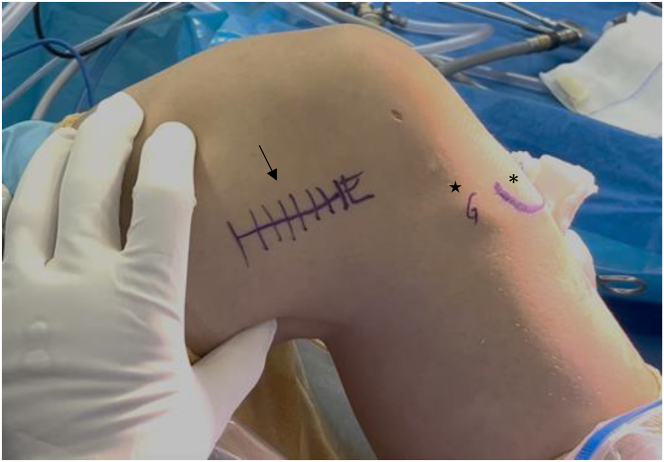
With the knee flexed at 90°, a 3- to 5-cm superolateral incision is made just proximal to the lateral epicondyle (right knee; black arrow). Anatomical landmarks including the tibial tuberosity (black asterisk) and Gerdy tubercle (black star) are identified.

A 3- to 5-cm longitudinal incision is made in the superolateral direction, just proximal to the lateral femoral epicondyle. The iliotibial band is dissected at its posterior third and retracted anteriorly (Fig 7).

Using electrocautery and scissors, the lateral thigh is dissected to reach the lateral intermuscular septum, which inserts into the lateral femoral condyle and separates the vastus lateralis muscle from the biceps femoris muscle. Once the lateral intermuscular septum is identified, the posterior aspect of the joint capsule can be reached.

**Fig 8:**
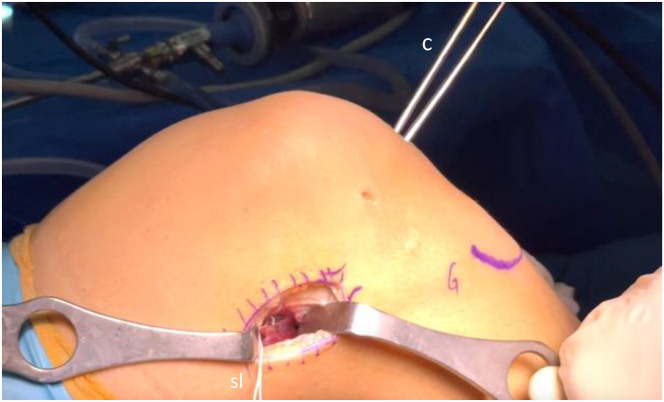
After opening the thin posterior layer of the knee capsule (right knee), a suture loop is passed from the superolateral approach through the over-the-top area to the anteromedial portal using a clamp. (c, clamp; sl, suture loop.)

A curved clamp is passed from the anteromedial portal to the notch, and its tip is positioned against the posterior capsule as proximally and as laterally as possible. Once the tip of the clamp is palpated from the posterolateral approach, it is pushed through the thin posterior layer of the knee capsule to open it to reach the previously prepared posterior space. A suture loop is then passed from the lateral side of the femur and retrieved through the anteromedial portal and later on through the tibial tunnel (Fig 8).

### Femoral Fixation in the OTT Zone

**Fig 9:**
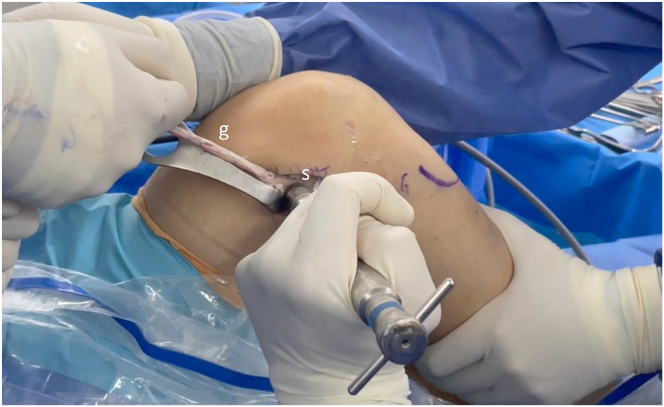
Fixation of the graft with 2 staples in the "over-the-top" area (right knee), while tensioning the graft with the knee flexed at 90°, the tibia in external rotation, and applying a posterior drawer. (g, graft; s, staple.)

A point just proximal to the lateral femoral epicondyle is identified and freed from soft tissue and periosteum. Once the spot for the fixation is prepared, the graft is tensioned pulling it in a proximal and ventral direction with the knee at 90°, the foot in neutral rotation while applying a posterior drawer. Two toothed staples are used parallels to the posterior femoral cortex to secure the graft to the lateral femoral cortex (Fig 9).

### Extra-articular Tenodesis

**Fig 10:**
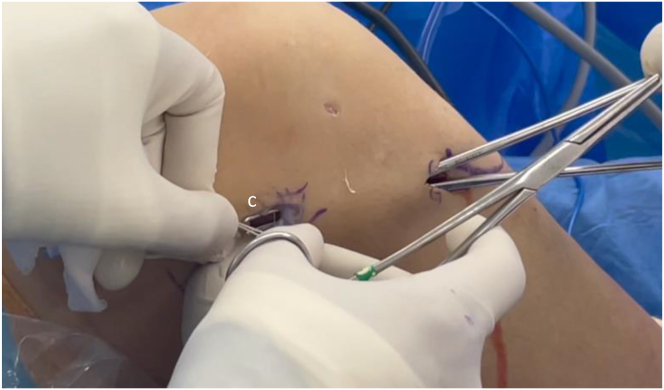
The graft is passed with a clamp (c) toward the Gerdy tubercle, beneath the fascia and over the lateral collateral ligament (right knee). With the knee flexed at 70° and the foot in neutral rotation, it is fixed to the Gerdy tubercle using a staple.

A 2-cm incision is made over the Gerdy tubercle. It is followed by a longitudinal incision on the anterolateral fascia in line with its fibers. A clamp is then inserted underneath the fascia and above the lateral collateral ligament while passing the graft towards Gerdy's tubercle (Fig 10). The graft is tensioned, and several flexion-extension cycles of the knee are performed. The graft is then fixed to the Gerdy tubercle with one staple, with the knee flexed at 70° with neutral foot rotation. A step-by-step description is summarized in Table 1. Pearls, pitfalls, and risks are listed in Table 2. Video 1 shows a step-by-step demonstration.

**Table 1:** Step-by-Step Augmented “Over-The-Top” Technique for Revision ACL Reconstruction

1. The patient is positioned supine under spinal or general anesthesia, with a tourniquet applied on the thigh and the leg supported in 90°of flexion using a lateral leg holder.
2. A 3-cm longitudinal incision is made medial to the anterior tibial tuberosity, and subcutaneous dissection is performed to expose the sartorius fascia over the hamstring tendons.
3. A transverse incision is made on the sartorius fascia to isolate the hamstring tendons, which are extracted using a 90° Semb dissector and open tendon stripper. It is essential to preserve their tibial insertion.
4. After removal of the muscular and soft tissue attachments, both hamstring tendons are sutured together with absorbable stitches to prepare for augmentation with the peroneus longus allograft.
5. The peroneus longus allograft is thawed in a saline solution containing vancomycin and then prepared by creating a sheet-like tubular structure using longitudinal incisions and a periosteal elevator.
6. The prepared peroneus longus tendon is wrapped circumferentially around the hamstring graft and secured with simple absorbable sutures followed by a continuous high-strength suture to reinforce the construct.
7. Arthroscopic exploration is performed through anteromedial, anterolateral, and accessory portals, clearing soft tissue from the over-the-top position of the femoral side and preparing the femoral tunnel with an ACL aiming guide.
8. On the tibial side, the tunnel is drilled over the native ACL origin with a diameter of the measured graft thickness, and a dilator is used to compress the tunnel walls, ensuring appropriate graft fit and secure integration with the femoral cortex.
9. A lateral femoral approach is performed by incising the skin, soft tissue and iliotibial band to access the posterior joint capsule, which is prepared for the graft passage.
10. A curved clamp is inserted arthroscopically through the notch and used to open the posterior thin capsule; a suture loop is then passed and retrieved to facilitate graft passage.
11. The femoral fixation point, located just proximal to the lateral femoral epicondyle, is cleared, and the graft is tensioned at 90° knee flexion and secured using two staples in-line with the posterior cortex.
12. Extra-articular tenodesis is completed by creating a tunnel to the Gerdy tubercle, tensioning the graft, verifying isometry, and fixing it with a staple at 70° flexion and neutral foot rotation.

ACL, anterior cruciate ligament.

**Table 2:** Pearl, Pitfalls, and Risks

Pearl
First, secure the semitendinosus and gracilis tendons together, followed by the sheet-like shape allograft, allows the graft to behave as a single unit.
When opening the posterolateral capsule, insert a finger between the popliteal neurovascular bundle and the Kelly clump to avoid neurovascular complications.
Use a 90° rasp to refresh the posterior portion of the lateral femoral condyle from the intercondylar notch to promote graft integration.
Apply graft traction from distal to proximal when performing femoral fixation, as well as a maximum posterior drawer force to obtain a well-tensioned graft.
Pitfalls and risks
Positioning the staples in the bone with excessive force may rupture the graft during the maximum tension phase.
Premature tendon cutting during harvesting, particularly if the grafts measure less than 22 cm, may compromise the possibility of performing the extra-articular tenodesis.
Failure to reach the capsular plane behind the lateral condyle without clearing out muscular fibers from the popliteus or gastrocnemius, will result in postoperative pain.

## Discussion

Single-stage ACL revision offers advantages over 2-stage surgery by reducing morbidity, infections, recovery time and lowering costs.^8^ Although 2-stage ACL revision allows for bone graft healing, the prolonged interval between surgeries increases the risk of additional intra-articular injuries due to joint instability.^9^

The OTT technique has some advantages because it avoids creating new femoral tunnels, reduces bone weakening, and facilitates graft integration. Above all, this surgical procedure allows us to do a single-stage surgery to resolve clinical cases in which it might otherwise be necessary to perform a 2-stage treatment due to the position or widening of the femoral tunnel.^3^ Moreover, preserving the tibial insertion of hamstring helps to prevent the initial avascular necrosis phase and ensures faster graft maturation, which is even more important in revision ACL setting.^10^ A recent meta-analysis by Vari et al.^11^ highlights that detached-hamstring grafts and hamstring grafts that are not detached produce equivalent outcomes. However, it seems that hamstring graft that are not detached tend to produce greater stability and lower graft failure rates. Moreover, Gupta et al.^12^ have recently demonstrated that tibial tunnel integration of the graft that are not detached occurs earlier than femoral tunnel integration, suggesting that the blood supply and remnant fiber muscles facilitate graft incorporation, as previously shown by Cuti et al.^13^

Lateral extra-articular tenodesis enhances rotational stability and protects the primary graft from re-rupture.^14^ However, concerns have been raised regarding its potential association with the development of osteoarthritis in the lateral knee compartment.^15^ Nevertheless, recent studies indicate that the OTT approach combined with LET does not increase lateral or patellofemoral osteoarthritis.^16^

Graft thickness plays a crucial role in ACL reconstruction. Studies indicate that grafts thicker than 8 mm provide greater biomechanical resistance and have lower failure rates.^17^ The described technique achieves a 2-mm increase in graft diameter. It ensures sufficient thickness for long-term stability, and it is able to fill a wider tibial tunnel even when leaving the hamstring insertion attached to the tibia.

Despite the numerous benefits of the technique described, some authors have raised concerns about the nonisometric function and nonanatomical position of the graft.^18^ However, several studies have shown that the OTT technique provides an almost anatomical orientation by placing the graft in the posterior region of the femoral condyle.^19^ A complete graft isometry is not a primary goal, as the native ACL is not an isometric structure.^20^ A list of the advantages and limitations of this procedure can be seen in Table 3.

**Table 3:** Advantages and Limitations

Advantages
Diminishes the risks and prolonged recovery associated with 2-stage revision ACLR.
Restores rotatory stability.
Cost-effective graft-fixation.
Limitations
Extra incision.
If primary ACLR was performed with hamstrings, an autograft is not possible.
Possible hardware-related symptoms on the femoral side.

ACLR, anterior cruciate ligament reconstruction.

In conclusion, the augmented OTT technique offers a reliable option for ACL revision, particularly when the primary surgery involved a tibial tunnel ≥8 mm. It facilitates correction of femoral tunnel malposition, enables grafts >8 mm in diameter, and preserves the native tibial insertion of the hamstrings.

## Disclosures

All authors (S.P., M.M., J.D., N.P., R.M.A., M.I., J.C.M.) declare that they have no known competing financial interests or personal relationships that could have appeared to influence the work reported in this paper.

## Supplementary Data

Video 1 Augmented over-the-top anterior cruciate ligament revision using peroneus longus allograft wrapped around preserved hamstrings.
